# Helminth species dependent effects on Th1 and Th17 cytokines in active tuberculosis patients and healthy community controls

**DOI:** 10.1371/journal.pntd.0010721

**Published:** 2022-08-17

**Authors:** Gezahegn Bewket, Amare Kiflie, Fitsumbrhan Tajebe, Ebba Abate, Thomas Schön, Robert Blomgran

**Affiliations:** 1 Department of Immunology and Molecular Biology, University of Gondar, Gondar, Ethiopia; 2 Ethiopian Public Health Institute, Addis Ababa, Ethiopia; 3 Division of Inflammation and Infection, Department of Biomedical and Clinical Sciences, Linköping University, Sweden; 4 Department of Infectious Diseases, Linköping University Hospital and Kalmar County Hospital, Linköping University, Sweden; Universidade Federal de Minas Gerais, BRAZIL

## Abstract

Despite that the impact of different helminth species is not well explored, the current dogma states that helminths affect the Th1/Th2 balance which in turn affects the risk of tuberculosis (TB) reactivation and severity of disease. We investigated the influence of helminth species on cytokine profiles including IL-17A in TB patients and healthy community controls (CCs). In total, 104 newly diagnosed pulmonary TB patients and 70 HIV negative and QuantiFERON negative CCs in Gondar, Ethiopia were included following helminth screening by stool microscopy. Plasma samples and *ex vivo* stimulation of peripheral blood mononuclear cells (PBMCs) with purified protein derivative (PPD) and Staphylococcus enterotoxin B (SEB) was used to determine cytokine profiles by cytometric bead array. In CCs, *Ascaris lumbricoides* or *Schistosoma mansoni* infections were associated with an impaired Th1-type response (IFN-gamma, IL-6 and TNF-alpha) in PBMCs mainly with SEB stimulations, whereas in TB patients only hookworm infection showed a similar pattern. Among CCs, the IL-17A response in PBMCs stimulated with SEB was higher only for *S*. *mansoni*, whereas in TB patients, the elevated systemic IL-17A plasma level was significantly suppressed in hookworm infected TB patients compared to patients without helminth coinfection. Following treatment of TB and helminth infection there was a general decrease in *ex vivio* IL-10 and TNF-alpha production in unstimulated, PPD or SEB stimulated PBMCs that was the most pronounced and significant in TB patients infected with *S*. *mansoni*, whereas the follow-up levels of IFN-gamma and IL-17A was significantly increased only in TB patients without helminth coinfection from PBMCs stimulated mainly with SEB. In summary, in addition to confirming helminth specific effects on the Th1/Th2 response before and after TB treatment, our novel finding is that IL-17A was impaired in helminth infected TB patients especially for hookworm, indicating a helminth species-specific immunoregulatory effect on IL-17A which needs to be further investigated.

## Author summary

Protection against tuberculosis (TB) requires a robust proinflammatory Th1-type immune response with cytokines like TNF and IFN-gamma being vital for cell recruitment and activation of microbicidal mechanisms, and granuloma formation to wall off the bacteria. Lately also the cytokine IL-17 was shown important for TB-protection. Helminths generally trigger a Th2-type immunity which suppresses Th1-type responses having consequences for TB reactivation and disease severity in endemic regions. To study helminths species-specific effects on the cytokine response for TB, which is not fully explored, we recruited patients with active pulmonary TB and healthy community controls (CCs) in Gondar, Ethiopia, and analyzed the Th1/Th2 cytokine profiles including IL-17A in plasma and from isolated and stimulated immune cells (PBMCs). In CCs confirmed not having latent TB we found that *Ascaris lumbricoides* or *Schistosoma mansoni* infection suppressed Th1 cytokines and IL-10 in PBMCs. In TB patients, on the other hand, only hookworm infection showed a similar pattern suppressing Th1 cytokines in PBMC, as well as suppressing the elevated systemic levels of IL-17A in plasma. Our findings show that helminths suppress the TB-protective cytokine output in a species-dependent manner whereby certain species possibly increase susceptibility to TB and others more strongly regulate immunity during TB disease.

## Introduction

Tuberculosis (TB) and helminth infections remain a global health challenge with *Mycobacterium tuberculosis* (Mtb) infection causing an estimated 10 million new TB cases yearly [1], and over 1.5 billion people in the world estimated to be infected with helminth species [2]. In addition to being major health problems as independent infections, coinfection with helminths and TB are common [3–5]. In Ethiopia, where TB and helminths are highly prevalent, helminth infections are more common in TB patients compared with community controls (40% versus 28%) [6].

The high association of helminth infections with active TB might be due to the helminth induced attenuation of Mtb-specific cell-mediated immune responses [7,8], which could increase the risk of Mtb infection as indicated by *Ascaris lumbricoides* and *Trichuris trichiura* infections being associated with tuberculin skin test (TST) positivity [9]. In addition, helminths like *Schistosoma mansoni*, might increase TB susceptibility by reducing the protective efficacy of Bacille Calmette-Guerin (BCG) vaccine through an attenuated Th1-type response [10,11]. Moreover, hookworm infection in TB patients was associated with the therapeutic failure of anti-TB drugs [12], which could contribute to the increased prevalence of TB. Helminths such as *Strongyloides stercoralis* [13] and *S*. *mansoni* [14] infections also increased TB disease severity.

Protection against TB is associated with a potent Th1 immune response characterized by the production of IL-2, IL-6, IL-12, IFN-gamma, and TNF-alpha [15,16]. The presence of multi-functional Th1 cells co-expressing IFN-gamma, TNF-alpha, and IL-2 in mice lungs correlated with protection against Mtb [17]. Experimental animal models indicate that IL-17 also has a protective role against Mtb infection [18–20], and a reduced number of IL-17 and IL-22 producing CD4+ T cells have been associated with active TB in man [21].

Helminth infections are associated with a Th2 dominant immune response with increased production of IL-4, IL-5, and IL-13 [22,23]. In addition, helminths induce regulatory T cells capable of producing IL-10 and TGF-beta which could suppress Th1- and Th17-, as well as Th2-type cellular responses [24,25]. It has been suggested that helminths affect the immune response against Mtb infections, through a Th2-dominated immune response, and the production of TGF-beta and IL-10 [26]. The frequency of regulatory T cells and production of IL-10 were increased in helminth infected TB patients compared to helminth negative TB patients [6], and deworming and anti-TB treatment of helminth infected TB patients decreased IL-10 production compared to helminth positive TB patients only treated with anti-TB drugs [27].

*S*. *stercoralis* and *Wuchereria bancrofti* infections, in active TB patients, suppressed the production of systemic IFN-gamma, TNF-alpha, IL-2, IL-17A and IL-17F [7]. These studies indicate that helminths impair TB protective immunity, however, the species-specific effects of different helminth species are rarely investigated [28,29] and need further investigation in areas where both TB and helminth infections are endemic. Therefore, our aim was to investigate the species-specific effect of helminths on the production of Th1, Th2, Th17 and the regulatory cytokine IL-10 in CCs and newly diagnosed pulmonary TB patients in Gondar, Ethiopia.

## Materials and methods

### Ethics statement

The study protocol was reviewed and received ethical clearance from the Ethics Review Board of University of Gondar, Ethiopia (O/V/P/RCS/05/1254/2016). Written informed consent was obtained from all study participants.

### Study participants

Newly diagnosed consecutive pulmonary tuberculosis patients were recruited at the Directly Observed Treatment Short-course (DOTS) Clinics of University of Gondar Specialized Hospital, Gondar Health Center, Maraki and Azezo Health Centers, from July 2016 to December 2018. The inclusion criteria were patients with age 18 to 65 years old with a smear positive result for acid-fast bacilli (smear positive TB) or Gene Xpert MTB/RIF (Xpert) positive result. The exclusion criteria were patients requiring hospital admission, MDR/XDR TB, HIV, pregnancy, ongoing treatment against helminths, intestinal protozoal infections, Diabetes Mellitus and other acute or chronic medical conditions, clinical signs or medical treatment indicating any concomitant infectious diseases other than TB. None of the study participants had symptomatic helminth infection during enrolment or follow-up.

All pulmonary TB patients received anti-TB treatment according to the national guideline [30]. Helminth positive TB patients and community controls (CCs) recruited for the study were offered anti-helminth treatment as part of the study protocol according to the Standard Treatment Guidelines [31]. *S*. *mansoni* infected TB patients and CCs received praziquantel (with a dose of 40 mg/kg given in two divided doses, 4–6 hours apart). TB patients and CCs infected with either *A*. *lumbricoides* or hookworm received 400mg albendazole in a single dose.

QuantiFERON-TB Gold In-Tube (QFT) (Qiagen, Australia) test was performed according to the instructions from the manufacturer to define latent tuberculosis infection (LTBI) in CCs. Only QFT negative CCs were included in the study and were from the same community as TB patients. QuantiFERON negative CCs who fulfilled the blood donation criteria were recruited either at the University of Gondar Specialized Hospital blood bank or from the surrounding areas of Gondar town. All CCs included in the study had a TB-score [32] value of ≤3 points and did not show any clinical signs or symptoms of clinical tuberculosis, helminth, or other infections.

### Clinical examination and follow-up

Pulmonary TB patients with or without helminths were followed for two months from the initiation of anti-TB treatment and/or receiving anti-helminthic drugs. Socio-demographic and clinical information were collected using a structured questionnaire. The TB score which has a value from 0 to 13 points, was assessed at baseline and at 2 month follow-up by measuring signs and symptoms suggestive for clinical TB (cough, haemoptysis, chest pain, dyspnea, night sweating, anemic conjunctivae, lung auscultation finding, tachycardia (≥100/min), temperature (≥37°C), body mass index (BMI) ≤18 kg/m2, BMI ≤16 kg/m2, mid-upper arm circumference (MUAC) ≤220 mm, and MUAC ≤200 mm), each contributing one point [32,33]. TB patients were classified into three disease severity classes depending on their TB score value: severity class I (SCI: 0–5 points), severity class II (SCII: 6–7 points) and severity class III (SCIII: 8–13) [32].

### HIV-screening

Screening for HIV was done at the voluntary counseling and testing clinic and at the Directly Observed Treatment Short- course (DOTS) clinic as part of provider initiated HIV counseling and testing programme (PIHCT) with Stat-Pak (HIV 1/2, Chembio Diagnostics systems Inc., USA), Unigold (Uni-Gold Recombigen HIV-1/2Trinity Biotech, USA) and SD-Bioline (SD BIOLINE HIV-1/2 3.0, USA). HIV positive patients were referred to the HIV clinics for further assessment and free antiretroviral treatment (ART) according to Ethiopian HIV/AIDS treatment guidelines and were not included in the study.

### Stool examination

From both TB patients and CCs, one stool sample was collected for the diagnosis of intestinal helminths. Stool examination and classification of participants either into helminth positive or negative was made using three techniques including direct stool microscopy [34], formol-ether stool concentration [34], and Kato-Katz techniques [34,35] performed by the same technician throughout the study. One in 10 slides was randomly selected and checked again blindly by another technician for quality control. The same stool sampling, examination, and classification strategy was used at 2 months follow-up.

### Sputum examination

Sputum samples were stained for acid-fast bacilli (AFB) and examined using the direct method and a fluorescent microscope at baseline and at 2 months follow-up on spot-spot sputum samples according to national guidelines [36]. GeneXpert MTB/RIF (Xpert) [37], a polymerase chain reaction (PCR) based test, was performed for all TB patients from the sputum samples stored at -20°C for a maximum of 3 to 4 months. The Xpert assay was used for simultaneous diagnosis of TB and rifampicin resistance.

### Isolation of peripheral blood mononuclear cells (PBMCs) from whole blood

Ten ml venous blood was collected into a heparinized tube from CCs and TB patients at baseline and 2 months follow-up. The blood samples were transported within two hours to the laboratory for PBMCs isolation. The blood was diluted with an equal volume of normal saline solution, layered on the top of LymphoPrep density gradient solution (Serumwerk, Bernburg AG, Oslo, Norway), and centrifuged at 800g for 30 minutes at 20°C with zero brake. After centrifugation, the interphase ring consisting of a mixture of mononuclear cells formed above the Lymphoprep solution and underneath the plasma. The plasma was stored at -80°C for cytokine analysis. The PBMCs were isolated as described previously [38], and counted using a Bürker chamber with 0.4% trypan blue solution (Sigma-Aldrich, Munich, Germany). All included samples had a cell viability above 75%, which was the cutoff point for the sample to be included for further cell stimulation.

### PBMC stimulation and harvesting of supernatant

Half a million PBMCs suspended with 500 μl RPMI 1640 medium were stimulated with either media alone as a negative control, Mtb-derived purified protein derivative (PPD; Statens Serum Institute, Copenhagen, Denmark) at a final concentration of 10 μg/ml to reveal Mtb-specific cytokine production, or stimulated with Staphylococcus enterotoxin B (SEB) antigen from Sigma Aldrich with a concentration of 5 μg/ml, as a polyclonal T cell stimuli to serve as a positive control for a strong T cell cytokine response. Stimulated cells were incubated at 37°C for 19h. After incubation, the supernatant was separated by centrifuging at 800 g for 5 minutes and stored at -80°C for later cytokine analysis using a cytometric bead array (CBA) assay.

### Cytometric bead array assay

Cytokine concentrations from the PBMC culture supernatants and plasma were analyzed using the Cytometric Bead Array (CBA) Human Th1/Th2/Th17 cytokine kit from BD Biosciences, San Diego, CA, USA, according to the manufacturer’s protocol. Beads coated with capture antibodies specific for IL-4, IL-6, IL-10, IFN-gamma, TNF-alpha or IL-17A was mixed with standards and unknown samples, and mixtures incubated with phycoerythrin conjugated detection antibodies for 3h. After incubation, beads were washed with wash buffer and acquired using a FACS Calibur flow cytometer (BD Biosciences) using Cell Quest acquisition software. A minimum of one thousand events were acquired from a single tube. The flow cytometric data was then analyzed by a Flowjo 7.6.5 (Tree Star, USA) software to calculate the cytokine concentration from the median fluorescence with the help of a standard curve using Microsoft excel.

### Statistical analysis

Data analysis was performed using Graph Pad Prism version 5.1 (Graph Pad Software, San Diego, CA). Continuous data are presented as mean ± SEM. Student’s t-test was applied to determine the difference between helminth negative and helminth positive (combined) groups. One-way ANOVA followed by Tukey’s multiple comparison test was used to determine the helminth species-specific effect within CCs groups and within pulmonary TB groups. Comparison between two time-point measurements on follow-up was made by paired Student’s t-test. Although this is mainly a descriptive study without previous data to base estimations of the immunological effects of helminths on what was investigated, we reasoned that with a helminth coinfection of about 30% in both groups in the area, 100 TB patients and 70 CCs in the control group were needed to reach at least 30 helminth-infected TB patients and 20 helminth-infected CCs. This was further estimated to result in a minimum of 6 subjects in each subgroup for the different helminth species which was considered a minimum to perform statistical analyses. For the TB patients and CCs to reach a power of 80% with an alpha of 0.05, we estimated that to show a difference between a pro-inflammatory cytokine response of 50% of the maximum (SEB stimulation) in the helminth negative group compared to a 20% response of maximum in the helminth positive patients there was a need to include at least 38 patients in each group. A p<0.05 was regarded as statistically significant.

## Results

### Socio-demographic and clinical characteristics of study participants

**Fig 1:**
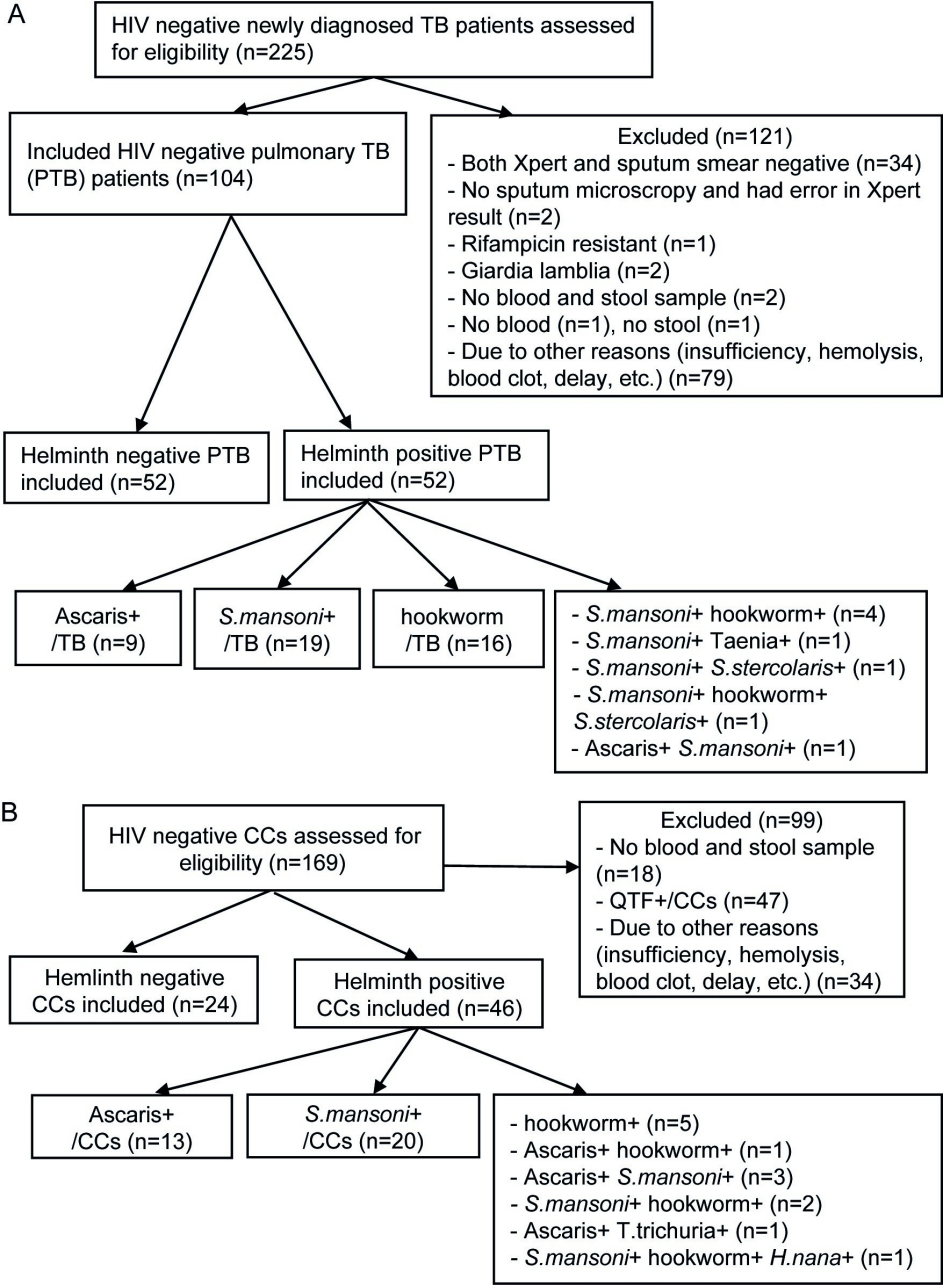
Flowchart of the inclusion process. (A) For pulmonary tuberculosis patients the study was conducted at four Directly Observed Treatment Short-Course Clinics of the University of Gondar Specialized Hospital, Gondar Health Center, Maraki and Azezo Health Centers, Ethiopia. (B) For healthy community controls.

We included a total of 70 CCs, 46 (65.7%) of them were helminth positive primarily with *S*. *mansoni* and *A*. *lumbricoides* either in single infection or in combination. Among the 104 included pulmonary TB patients, 52 (50%) were helminth infected with the majority of them positive for *S*. *mansoni*, hookworm and *A*. *lumbricoides* (Fig 1). Subjects having only single infections either with *S*. *mansoni*, hookworm, or *A*. *lumbricoides* were included for species-specific analysis. No baseline differences in sex distribution, mean age, and BMI were found in CCs. The mean TB score of hookworm positive TB patients was significantly lower compared to helminth negative TB patients (P< 0.009) (Table 1).

**Table 1:** Demographic characteristics and TB score values of CCs and TB patients.

		Hel-/CCs	Hel+/CCs	Asc+/CCs	*S*.*m*+/CCs	HW+/CCs	P^c^	Hel-/TB	Hel+/TB	Asc+/TB	*S*.*m*+/TB	HW+/TB	P^TB^
N		24	46	13	20	5		52	52	9	19	16	NS
Sex %	M	66.7	47.8	30.8	65	3	NA	53.1	75	77.8	83.3	75	NA
	F	33.3	52.2	69.2	35	2	NA	46.9	25	22.2	16.7	25	NA
Age (years), mean ±SD		26.6 ±2.9	26.8 ±9.3	31.6 ±12.5	24.7 ±7.7	26.8 ±2.6	NS	31.7 ±14.7	29.7 ±12.8	24.9 ±8.2	29.6 ±12.7	30.9 ±12.6	NS
BMI (Kg/m^2^),mean ±SD		22.4 ±3.0	20.7 ±3 .5	20.4 ±4.4	20.9 ±3.5	21.8 ±1.9	NS	18.0 ±2.1	18.2 ±1.8	18.1 ±1.6	18.3 ±2.4	18.0 ±1.5	NS
TB score, mean ±SD		NA	NA	NA	NA	NA	NA	7.1 ±2.7	5.9 ±2.1	5.8 ±2.1	7.0 ±2.4	4.7 ±1.6	P^tb^HW < 0.009

SD: Standard deviation; BMI: Body mass index; Kg/m^2^: kilogram per meter square; CCs: community controls; TB: pulmonary tuberculosis; Hel-, helminth negative, Hel+, helminth positive; Asc+: *A*. *lumbricoides* positive; S.m+: *Schistosoma mansoni* positive; HW+: hookworm positive; P^c^: p-values of Hel+/CCs groups compared to Hel-/CCs; P^TB^: p-values of Hel+/TB groups compared to Hel-/TB; P^tb^HW: p-value of hookworm+/TB versus Hel-/TB; NA: not analyzed; NS: no significant p-values between indicated Hel+ and Hel- groups. Only significant p-values are displayed.

### The levels of Th1 cytokines and IL-10 are lower but IL-17A is higher in helminth infected CCs

**Fig 2:**
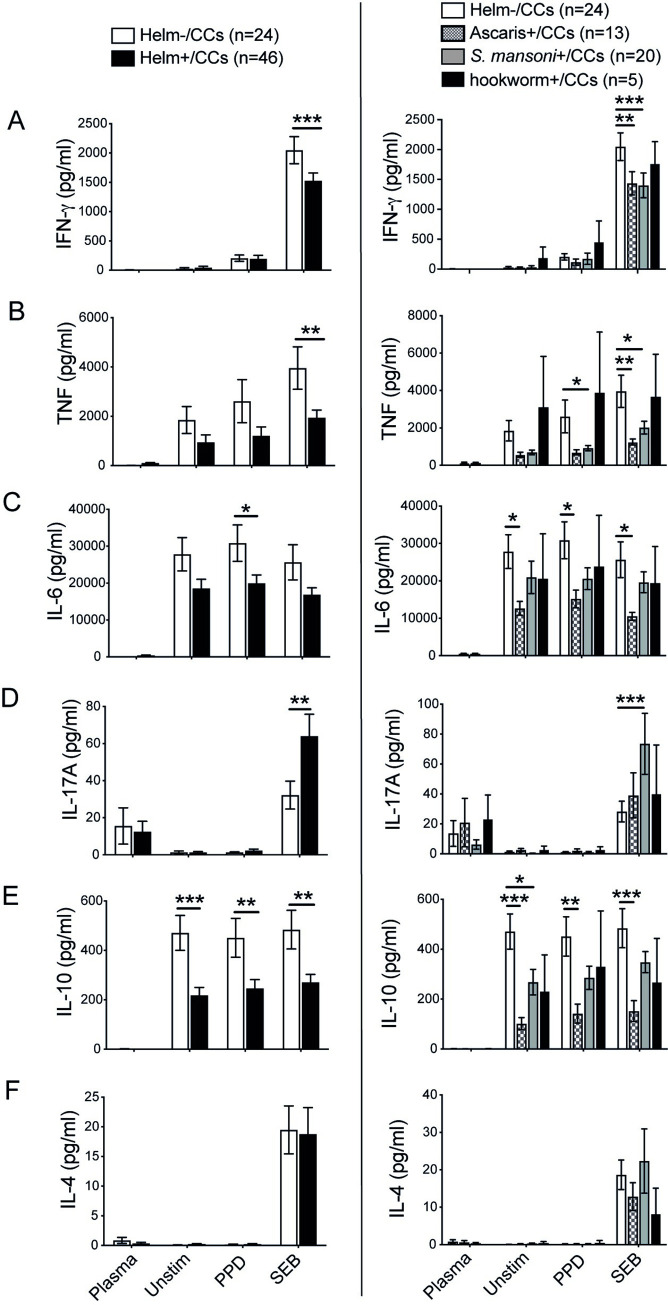
Community controls with helminth infection show increased IL-17A levels as well as reduced levels of Th1 cytokine and IL-10. PBMCs isolated from QFT negative CCs with or without helminth infection were stimulated with media alone (Unstim), PPD or SEB and cultured for 19h at 37°C, and PBMC supernatants harvested. The level of IFN-gamma, TNF-alpha, IL-6, IL-17A, IL-10 and IL-4 were determined in plasma and from PBMC supernatants using cytometric bead array. Concentration of IFN-gamma (A), TNF-alpha (B), IL-6 (C), IL-17A (D), IL-10 (E) and IL-4 (F) in helminth negative (Helm-) and helminth positive (Helm+; all helminth positive combined) CCs (left), and in subgroup analysis of different helminth species (right). Data are presented as mean ± SEM. n = 24 for Helm-/CCs and n = 46 for Helm+/CCs, and n = 13 for *Ascaris lumbricoides*+/CCs, n = 20 *Schistosoma mansoni*+/CCs, and n = 5 hookworm+/CCs. Statistical analysis was performed using Student’s t-test for comparing Helm-/CCs versus Helm+/CCs. One-way ANOVA with Tukey’s multiple comparison test was used for examining the helminth species-specific effect. The limited hookworm+/CCs, n = 5, in the helminth species-specific analysis to the right were not included in the statistical analysis. *, p<0.05; **, p<0.01, ***, p< 0.001.

To determine the impact of helminth infection on the immune response in CCs, we first grouped all helminths into one as Helm+/CCs and measured the concentration of Th1 (IFN-gamma, TNF-alpha, IL-6), Th17 (IL-17A), regulatory (IL-10) and a Th2 (IL-4) cytokines in plasma and supernatants harvested from unstimulated, PPD or SEB stimulated PBMCs. The general trend in the lower level of Th1 cytokines in unstimulated, PPD and SEB stimulated PBMCs were observed in Helm+/CCs compared to Helm-/CCs. The production of IFN-gamma and TNF-alpha were significantly reduced in SEB stimulated PBMCs while the level of IL-6 was significantly lower in PPD stimulated PBMCs of Helm+/CCs compared to Helm-/CCs. The production of IL-10 was also significantly reduced in Helm+/CCs compared to Helm-/CCs from PBMCs with no stimulation or with PPD or SEB stimulations, whereas the level of IL-17A was significantly higher in Helm+/CCs compared to Helm-/CCs in SEB stimulation (Fig 2A–2E).

The helminth species-specific analysis showed that CCs infected by *A*. *lumbricoides* or *S*. *mansoni* exhibited a significantly lower level of IFN-gamma and TNF-alpha mainly in SEB stimulated PBMCs. For IL-6 and IL-10, *A*. *lumbricoides* infected CCs, had significantly lower levels of IL-6 and IL-10 compared to Helm-/CCs for all PBMCs (unstimulated as well as stimulated), whereas *S*. *mansoni* only significantly lowered IL-10 in unstimulated PBMCs. The level of IL-17A that was significantly higher in SEB stimulated PBMCs in Helm+/CCs compared to Helm-/CCs, was only significantly associated with *S*. *mansoni* infection in the helminth species-specific analysis (Fig 2A–2E). As single hookworm infected CCs were very few (n = 5), they are shown in Fig 2 merely for illustrative purposes but not included in statistical analysis or discussed further in this manuscript.

### Hookworm infection in TB patients is associated with reduced levels of IFN-gamma, IL-6 and IL-17A

**Fig 3:**
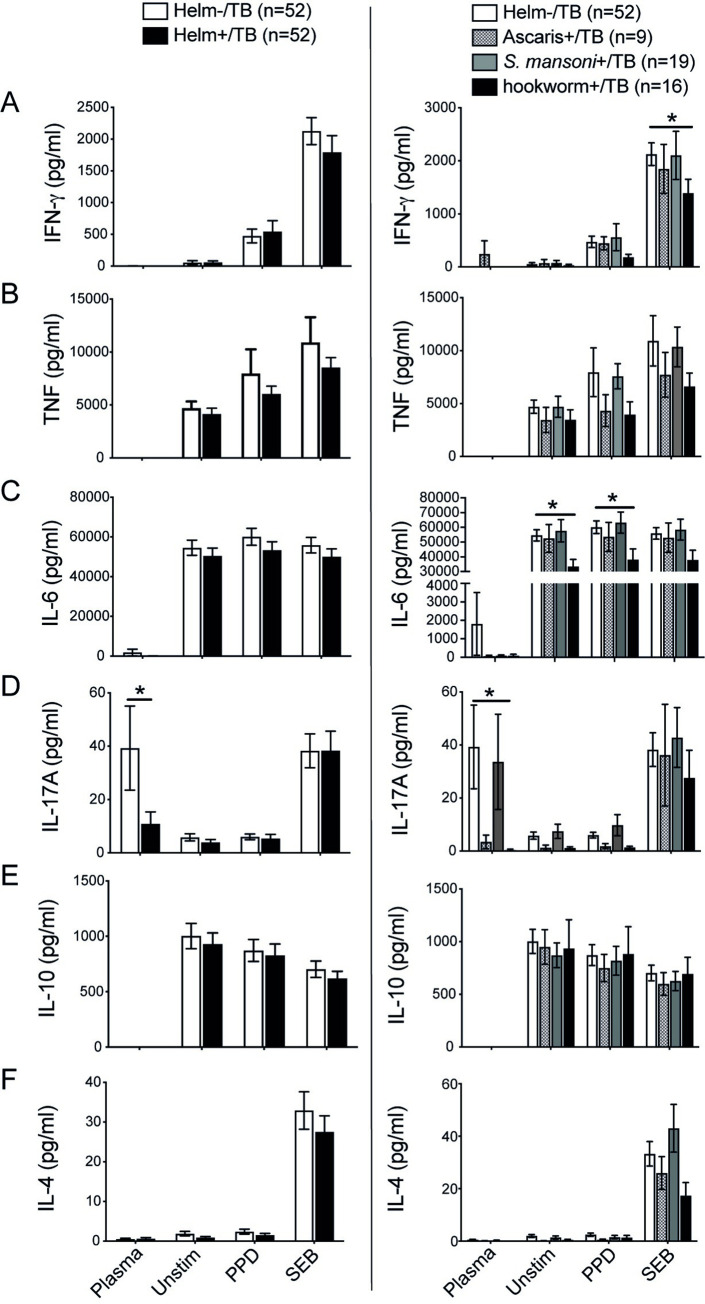
Helminth infection reduced Th1 and Th17 cytokines in TB patients in a species-dependent pattern. PBMCs isolated from TB patients with or without helminths were stimulated with media alone (Unstim), PPD or SEB and cultured for 19h at 37°C, and PBMC supernatants harvested. The level of IFN-gamma, TNF-alpha, IL-6, IL-17A, IL-10 and IL-4 were determined in plasma and from PBMC supernatants using cytometric bead array. Concentration of IFN-gamma (A), TNF-alpha (B), IL-6 (C), IL-17A (D), IL-10 (E) and IL-4 (F) in helminth negative (Helm-) and helminth positive (Helm +; all helminth positive combined) TB patients (left), and in the subgroup analysis of different helminth species (right). Data are presented as mean ± SEM. n = 52 for both Helm- and Helm+ TB groups, and n = 9/19/16 for *A*. *lumbricoides*+, *S*. *mansoni*+ and hookworm+ TB patients, respectively. Statistical analysis was performed using Student’s t-test for comparing Helm-/TB versus Helm+/TB. One-way ANOVA with Tukey’s multiple comparison test was used for examining the helminth species-specific effect. *, p<0.05.

When all helminth positive patients were grouped together as Helm+/TB, the level of IFN-gamma, TNF-alpha, IL-6, IL-10 and IL-4 was not statistically different between helminth negative and helminth positive TB groups (Fig 3A–3F). However, the production of IFN-gamma was significantly higher in Helm-/TB patients compared with Helm+/CCs. The level of proinflammatory cytokines (TNF-alpha and IL-6) and a regulatory cytokine IL-10 were also significantly higher in both TB groups (in Helm-/TB and Helm+/TB) regardless of helminth infection status compared to Helm-/CCs or Helm+/CCs in unstimulated, PPD stimulated or SEB stimulated PBMCs (S1 Fig), indicating that the level of inflammation was high during active TB and that higher level of IL-10 was concomitantly produced to prevent the excessive inflammation. Plasma IL-17A production was significantly lower in the Helm+/TB group compared to Helm-/TB group (p < 0.05) (Fig 3D). The level of IL-17A in plasma was also significantly lower in Helm+/CCs compared to Helm-/TB (p<0.01), while there was no significant difference in the plasma IL-17A between Helm-/CCs versus Helm+/CCs, Helm-/CCs versus Helm-/TB nor between Helm+/CCs versus Helm+/TB (S1 Fig). This suggests that TB does not significantly affect the production of plasma IL-17A rather helminths had an association with the reduced production of systemic IL-17A in the plasma of TB patients but not in CCs.

In the helminth species-specific analysis, IFN-gamma was significantly lower in hookworm+/TB compared with Helm-/TB (p < 0.05) in SEB stimulated PBMCs, showing the same trend in PPD-stimulated cells (Fig 3A). Compared to Helm-/TB, hookworm infection also showed significantly lower production of IL-6 in unstimulated (p<0.05) and PPD stimulated (p<0.05) PBMCs (Fig 3C). The helminth species-specific analysis further showed that the significantly reduced IL-17A production observed in the plasma of the Helm+/TB compared with Helm-/TB, was highly attributed to hookworm (p<0.05), and to *A*. *lumbricoides* although without reaching statistical significance (Fig 3D).

### Dynamics in cytokine responses after 2-months follow-up of TB patients in relation to anti-helminth therapy

**Fig 4:**
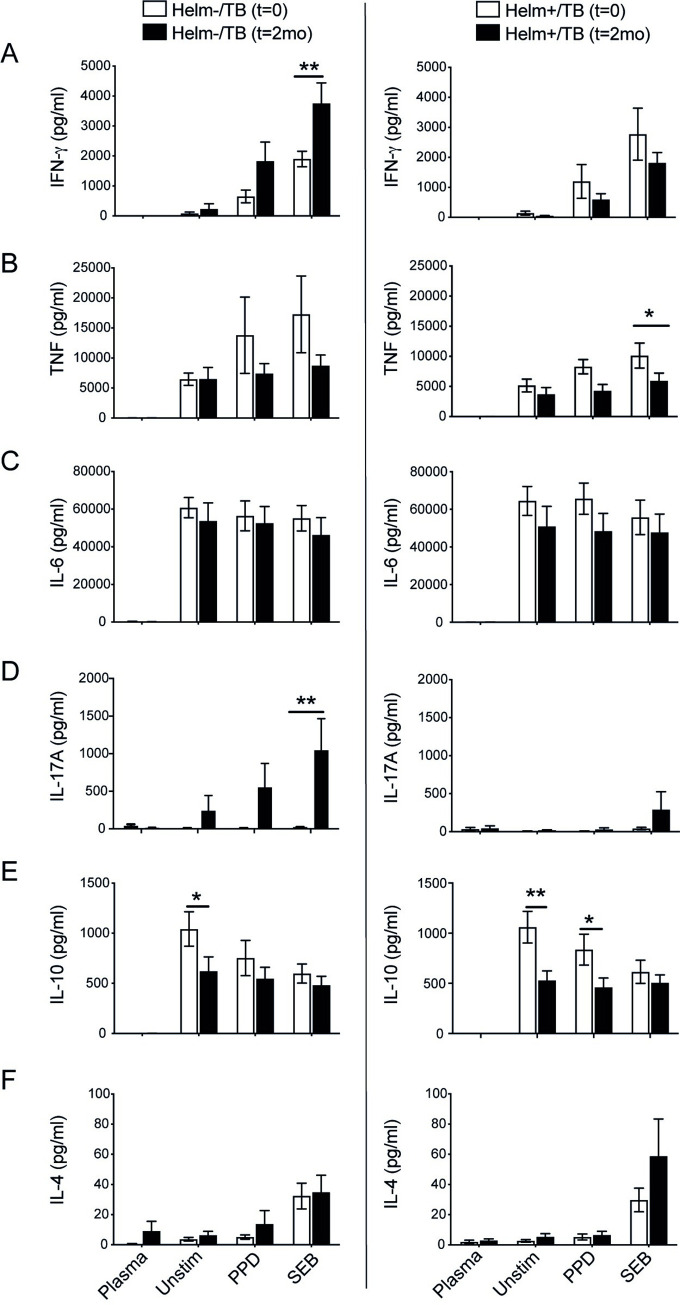
Concentration of Th1, Th2, Th17 and regulatory cytokines in TB patients with or without helminths at baseline and 2 months follow-up of anti-TB treatment. PBMCs isolated from TB patients with or without helminths at baseline (t = 0) and at 2 months follow-up (t = 2mo), were stimulated with media alone (Unstim), PPD or SEB, and cultured for 19h at 37°C, and PBMC supernatants harvested. Helminth coinfected TB patients received anti-helminthic treatment immediately after the baseline enrolment. The level of IFN-gamma, TNF-alpha, IL-6, IL-17A, IL-10 and IL-4 were determined in plasma and from PBMC supernatants using cytometric bead array. Concentration of IFN-gamma (A), TNF-alpha (B), IL-6 (C), IL-17A (D), IL-10 (E) and IL-4 (F) at (t = 0) and (t = 2mo) in helminth negative (Helm-/TB) patients (left), and in helminth positive (Helm+/TB) patients (right). Data presented as mean ± SEM. n = 18 for Helm-/TB and n = 14 for Helm+/TB. Statistical comparisons were made using paired Student’s t-test. *, p<0.05; **, p<0.01.

For the assessment of the immune response in TB patients with or without helminth coinfection after 2-months follow-up, n = 18 helminth negative TB patients, and 14 helminth positive TB patients treated with anti-helminth therapy (praziquantel for Schistosomiasis in all cases) were available for analysis. Helm+/TB patients were infected with either *S*. *mansoni* only (n = 7) and *S*. *mansoni* along with other helminth coinfections (n = 7). We analyzed the level of IFN-gamma, TNF-alpha, IL-6, IL-17A, IL-10 and IL-4 at inclusion before start of anti-TB treatment and anti-helminthic drugs in helminth positive patients (t = 0) and at the 2 months follow-up (t = 2mo). The production of IFN-gamma was increased in Helm-/TB after 2-months follow-up (p < 0.05) with SEB stimulation. However, there was no significant difference in the level of IFN-gamma in Helm+/TB groups between the baseline (t = 0) and 2-months follow-up (Fig 4A).

TNF-alpha production in Helm+/TB at t = 2mo, significantly decreased with SEB stimulation (p < 0.05) (Fig 4B). The IL-17A response was instead significantly increased at t = 2mo only in the Helm-/TB group with SEB stimulation (p < 0.05).

There was a significant decline of IL-10 in unstimulated PBMCs for both Helm-/TB (p < 0.05) and Helm+/TB (p < 0.01) at t = 2mo compared with their respective groups at t = 0. Moreover, the level of IL-10 in Helm+/TB after PPD stimulation had also significantly declined at t = 2mo (p < 0.05) (Fig 4E). IL-4 production was not significantly different in either Helm-/TB or Helm+/TB between baseline (t = 0) and 2-months follow-up (t = 2mo) (Fig 4F).

### Helminth infected TB patients with an intermediate disease score had a distinct cytokine response

**Fig 5:**
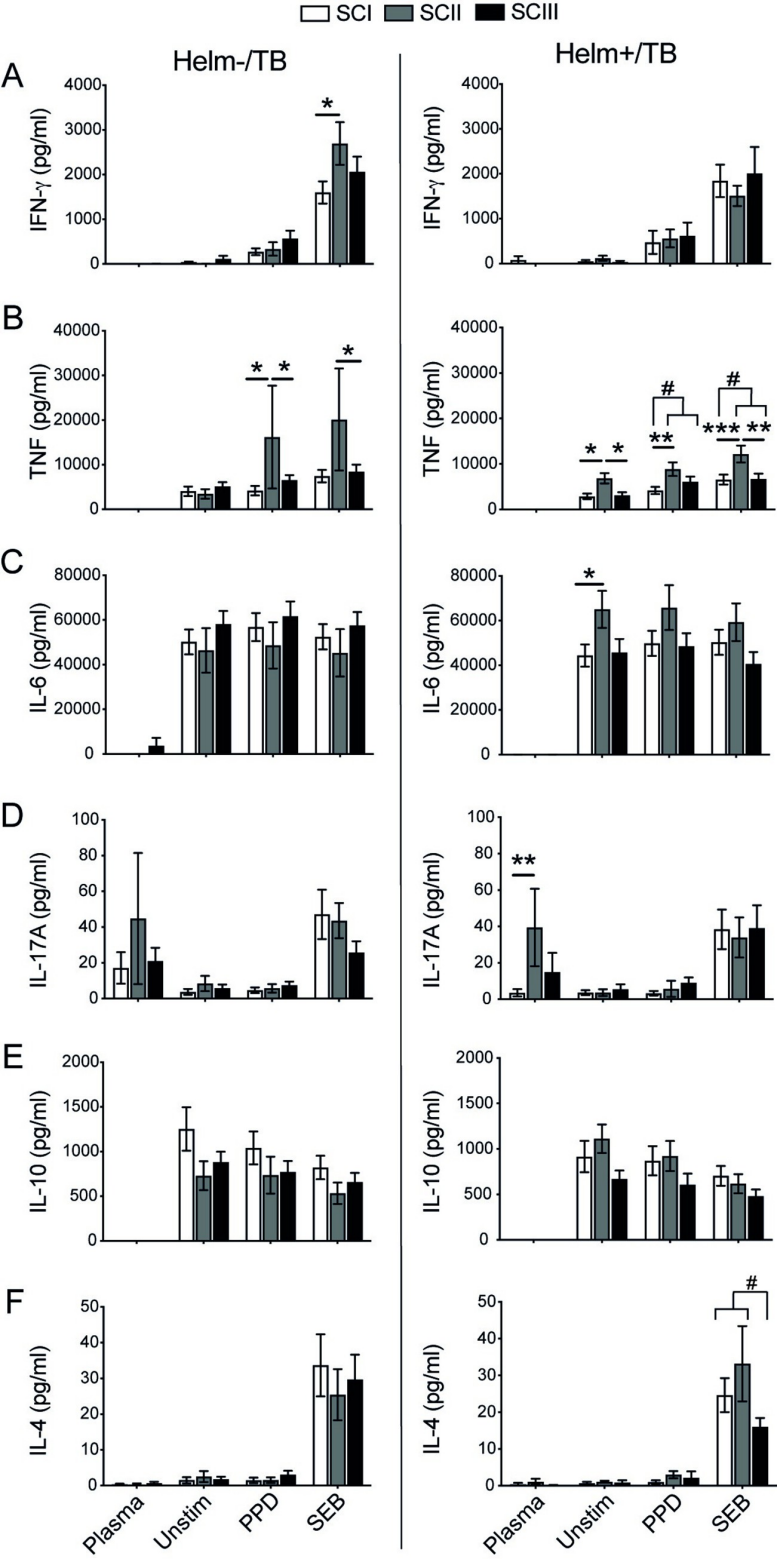
TB disease severity and its association with Th1, Th2, Th17 and regulatory cytokines production in TB patients with or without helminth infection. Concentration of cytokines in TB patients based on their helminth status and disease severity using TB score to classify patients into the three disease severity classes (SCI, SCII, SCIII). The concentration of IFN-gamma, TNF-alpha, IL-6, IL-17A, IL-10 and IL-4 were determined in plasma and from PBMC supernatants using cytometric bead array. Concentration of IFN-gamma (A), TNF-alpha (B), IL-6 (C), IL-17A (D), IL-10 (E) and IL-4 (F) in helminth negative (Helm-/TB) (left) and helminth positive (Helm+/TB) TB patients (right) with different disease severity classes. Data presented as mean ± SEM, and from TB patients with SCI (n = 20/27), SCII (10/15), and SCIII (25/16), that were Helm-/Helm+, respectively. Statistical analysis for the differences between the three disease severity classes performed using one-way ANOVA followed by Tukey’s multiple comparison test and differences shown by asterisks (*), and brackets indicate the additional analysis after combining SCII+SCIII in B and after combining SCI+SCII in F and significance showed using number sign (#). *^/#^, p<0.05; **, p<0.01, ***, p<0.001.

To assess whether TB disease severity classified into three classes (SCI, SCII, and SCIII) had an association with the production of IFN-gamma, TNF-alpha, IL-6, IL-17A, IL-10, and IL-4, we analyzed the level of each cytokine in both helminth infected and helminth negative TB patients based on their severity classes, separately. In both Helm-/TB and Helm+/TB groups with SCII, TNF-alpha production was higher compared with SCI and SCIII of their respective groups. Analysis made by combining the intermediate (SCII) and severe (SCIII) disease scores into one group (SCII-III) was performed to allow comparison of these patients with more common disease activity manifestations of TB to those with mild TB (SCI). This showed that only TNF-alpha was significantly higher in SCII-SCIII compared to SCI for Helm+/TB groups of both PPD and SEB stimulated PBMCs (Fig 5B). Moreover, analysis was also made by combining the minimal (SCI) and intermediate (SCII) disease scores into one group (SCI-II) to compare with the more severe (SCIII) diseased groups, and no significant difference except for the IL-4 response of PBMCs from helminth positive TB patients stimulated with SEB was found. In Helm+/TB groups, the level of IL-6 in unstimulated PBMCs and the systemic IL-17A was significantly higher in SCII compared with SCI (Fig 5C and 5D).

Overall this analysis revealed a distinct cytokine pattern for patients with intermediate disease severity, where the production of TNF-alpha, IL-6, and IL-17A was significantly increased in helminth coinfected TB patients with SCII, and IFN-gamma and TNF-alpha significantly increased in SCII of TB patients with no helminth infection.

## Discussion

Many previous studies assessing the effect of helminths on the immune response of TB patients and healthy controls show contrasting results and are based either on analyzing at the group level of different helminth species [6,27] or a single helminth species [39–41]. In this study, we investigated the species-specific effect of three helminths prevalent at the same endemic site on the level of Th1, Th2 and Th17 response and the regulatory cytokine IL-10. A novel observation is that the systemic IL-17 plasma level was significantly reduced in helminth infected TB patients, particularly among hookworm infected TB patients. Additionally, the 2-month follow-up of *S*. *mansoni* positive TB patients showed no improvement in the IFN-gamma or IL-17A producing capacity of PBMCs in response to SEB, which was the case for helminth negative TB patients.

Although it needs further validation by a cohort with larger sample size, we found that *A*. *lumbricoides* or *S*. *mansoni* infection in CCs had lower levels of Th1 cytokines (IFN-gamma, TNF-alpha and IL-6) from stimulated PBMCs mainly with SEB than helminth negative CCs, whereas only *S*. *mansoni* was associated with increased IL-17A from PBMCs upon *ex vivo* stimulation with SEB. In TB patients, on the other hand, there were lower Th1 and Th17 responses mainly during hookworm infection. We previously demonstrated that helminth-mediated expansion of non-classical monocytes in TB patients was species-dependent [29]. Thus, although the sample size for a species subgroup analysis was low, our data shows that helminth-induced modulation of cytokine response is affected in a species-specific manner. Only hookworm significantly reduced Th1 cytokine responses in TB patients, while in CCs, production of Th1 cytokines from *ex vivo* stimulated PBMCs mainly with SEB were reduced by *A*. *lumbricoides* or *S*. *mansoni* infection. Moreover, we demonstrated that the IL-17A response by SEB stimulated PBMCs was higher in *S*. *mansoni* infected CCs, whilst hookworm infection in TB patients instead significantly reduced plasma IL-17A. When helminth positive TB patients were analyzed as a group combining all helminth infections, the IFN-gamma response was neither significantly affected by helminths nor had an association with the level of disease severity.

In a mice experimental study of *Ascaris suum* infection, multiple exposure to larvae elicited an increased production of IL-17A with simultaneous increased production of Th2 cytokines (IL-4, IL-5), proinflammatory cytokines (IL-6, TNF-alpha) and of IL-10 compared to the single infected or non-infected mice [42]. However, in an experimental infection of humans with *Ascaris suum*, the level of IL-17A was decreased while IL-4 and TGF-beta were increased [43]. We found that natural helminth infection with *S. mansoni* but not Ascaris exhibited an increased IL-17A response. In line with that, IL-17A production has previously been shown to be significantly increased in mice with *Schistosoma* infection [44], which contributed to severe immunopathology [45,46], granulomatous inflammation and liver damage [47,48] in mice.

However, the TB-associated elevation in systemic plasma IL-17A was significantly suppressed in hookworm and to a lesser extent but not significantly in *A*. *lumbricoides* infected TB patients which is a novel finding. Although IL-17A is produced mainly by Th17 cells, innate immune cells in the peripheral blood such as neutrophils and eosinophils, and various mucosal and tissue associated innate immune cells such as macrophages and mast cells, together with other T-cell subsets such as gamma delta T cells, and mucosal-associated invariant T cells also produce IL-17A [49,50]. The lowered production of plasma IL-17A during hookworm and *A*. *lumbricoides* infections in active TB patients could thus be a suppression of the IL-17A production in any or several of these effector cells that directly or indirectly (by releasing IL-17A) are involved in TB protective immunity. For example, IL-17A production increases in Mtb infected neutrophils and autocrine IL-17A-production by neutrophils was vital for inhibiting Mtb growth by neutrophils through increased reactive oxygen species production and their migration to the site of infection [51]. Further, vaccination of mice with early secreted antigenic target protein 6 (ESAT-6) from Mtb triggered an early IL-17A response that enhanced the migration of IFN-gamma producing CD4+ T cells to the lungs and restricted Mtb growth [20]. This indicates that the reduction in IL-17A production by helminths may be involved in the host immune control of Mtb growth in the lungs of TB patients through several T-cell-independent pathways.

Th1 cytokines, primarily IFN-gamma and TNF-alpha polarize macrophages to a classically activated (M1) subtype, having high Mtb killing capacity, while Th2 cytokines lead to an alternatively activated (M2) subtype that favors intracellular Mtb multiplication [52,53]. Moreover, IFN-gamma and TNF-alpha are also involved in the formation of granuloma, in which Mtb can be contained [54]. In a mice, IL-6 has been shown to be involved in the initiation of early inflammatory responses and in reducing early mycobacterial load [55]. Hence, the observed impairment of Th1 cytokine responses, during *A*. *lumbricoides* or *S*. *mansoni* infection in CCs, suggests that helminths could lead to enhanced susceptibility for TB infection, although this needs to be shown in large epidemiological studies. However, in TB patients, the Th1 cytokines were significantly lowered only during hookworm infection. The possible explanation might be a presence of a low number of activated CD4+ and CD8+ T cells and an increased number of exhausted CD4+ T cells during hookworm infection in TB [56]. This is partly supported by the significantly reduced disease severity of hookworm infected TB patients at baseline indicating an attenuated pro-inflammatory response (Table 1). Although there was a trend of non-significant decrease in the Th1 and Th17 cytokine response in hookworm infected TB patients compared with *A*. *lumbricoides* or *S*. *mansoni* infected TB patients, there were no significant differences in the interspecific influence of cytokine production between these helminths in CCs or TB patients. Unlike hookworm, *A*. *lumbricoides* or *S*. *mansoni* infection in TB was not associated with significantly reduced Th1 cytokines. In a previous study, *A*. *lumbricoides* infection in TB patients did not significantly affect the production of Th1, Th2, and Th17 cytokines [57]. *S*. *mansoni* infection also did not affect the capacity of CD4 T cells to produce Th1 and Th2 cytokines in active TB patients and LTB infected individuals [58]. We recently showed that only hookworm but not *A*. *lumbricoides* or *S*. *mansoni* infection significantly increased the frequency of TGF-beta producing regulatory T cells in TB patients [28], which might partly explain the significantly downregulated production of Th1 cytokines only in hookworm infected TB patients. The differential species-specific immunological impact of different helminths might be due to the presence of different classes of helminth-derived immune modulatory molecules with various immune manipulation mechanisms [59].

As a regulatory cytokine, IL-10 is involved in preventing excessive inflammation [60], and in TB patients receiving anti-TB treatment the gradual reduction in pulmonary inflammatory lesions is mirrored by a reduction in IL-10 mRNA in isolated PBMCs [61]. In our study, although there was a 2-fold increased production of IL-10 in helminth negative TB patients compared with helminth negative CCs, this *ex vivo* IL-10 production was further reduced in helminth positive CCs where the pro-inflammatory cytokine production was concomitantly low. Based on these results we hypothesize that IL-10 is not directly influencing the expression of other major cytokines included in our study but rather align with the general inflammatory environment to prevent the effects of excessive Th1 inflammation.

After the 2 months follow-up of anti-TB and/or anti-helminthic treatment, IFN-gamma and IL-17A production was increased to statistically significance only in helminth negative TB groups from PBMCs stimulated with SEB. This indicates that anti-TB treatment after the intensive treatment phase could restore Th1 and Th17 response in helminth negative TB, but not in *S*. *mansoni* positive groups even after 2 months of receiving anti-helminthic drugs which might suggest a longer lasting effect of *S*. *mansoni* infection on TB protective immunity. Similarly, in previous studies, IFN-gamma production was significantly improved at 2 months and 6 months follow-up of TB treatment [62], and IL-17 increased at 6 months of TB treatment [63], which further supports our findings that TB protective immunity could be restored with anti-TB treatment by improving IFN-gamma and IL-17A cytokine responses in helminth negative TB patients, and that anti-TB treatment-induced improvement of the Th1 and Th17 response could be altered by certain helminths species such as *S*. *mansoni*.

Although, there was no difference between the baselines IL-10 level of helminth negative and helminth (mainly *S mansoni*) positive TB patients, IL-10 production by PBMCs with or without *ex vivo* PPD or SEB stimulation showed a decreased trend in helminth negative and more profoundly decreased in *S*. *mansoni* positive TB groups at 2 months follow-up. In a previous follow-up study, IL-10 significantly declined in helminth infected TB patients mainly with *A*. *lumbricoides* treated with albendazole compared with the placebo [27]. The observed decreased trend in TNF-alpha in helminth negative TB was also more strongly pronounced and significant in *S*. *mansoni* positive TB groups with SEB at 2 months follow-up, which might indicate that treatment of *S*. *mansoni* positive TB patients with anti-TB and anti-helminths could cause the rapid and more enhanced resolution of inflammation.

Although we observed the same TB-specific response (PPD as stimuli), the major significant changes to helminth infection we demonstrated were related to a decreased strong T cell response (with SEB as stimuli), which indicated that the T cell response is impaired as a result of helminth infection.

A limitation in our study was that the sample size for helminth species subgroup analysis was small especially in the *A*. *lumbricoides* positive groups. The helminth egg intensity in the majority of helminth positive CCs and TB patients were low [29] which might have an impact to verify the immunological consequence of helminths but is also explained by the procedure to exclude symptomatic helminth infection. Hookworm single positive CCs were very limited in this study. Thus, we could not confirm how hookworm affected the cytokine responses in CCs. Furthermore, all TB patients included in the follow-up were *S*. *mansoni* infected, which might not have the same immunological effect as *A*. *lumbricoides* or hookworm, after 2 months of anti-TB and anti-helminthic treatment.

In summary, the findings indicate that helminths affected the production of Th1 and Th17 cytokines differently in CCs and TB patients and that was also helminth species dependent. Further, at 2 months follow-up, IL-10 and TNF-alpha exhibited a decreased trend in helminth negative TB that was more pronounced and significant in TB patients infected with *S*. *mansoni*, indicating that anti-helminthic therapy could be considered during TB treatment in areas where helminths are common for the better control of disease progression and enhanced resolution of inflammation. Further clinical studies with large cohorts are warranted to further elucidate the species-specific impact of helminths on host immunity and clinical outcome.

## Supporting information

S1 Fig Proinflammatory cytokines and IL-10 are increased in TB patients regardless of helminth infection compared with CCs. PBMCs isolated from TB patients or CCs with or without helminths were stimulated with media alone (Unstim), PPD or SEB and cultured for 19h at 37°C, and PBMC supernatants harvested. The level of IFN-gamma, TNF-alpha, IL-6, IL-17A, IL-10 and IL-4 were determined in plasma and from PBMC supernatants using cytometric bead array. Concentration of IFN-gamma (left) and TNF-alpha (right) (A), IL-6 (left) and IL-17A (right) (B), IL-4 (left) and IL-10 (right). Overall, there were increased levels of pro-inflammatory cytokines in TB patients compared to CCs. Regarding IL-17A, only the Helm-/TB groups show increased levels in plasma whereas Helm+/TB patients were suppressed to the same level as the CC groups (p<0.01). Data are presented as mean ± SEM. n = 24 for Helm-/CCs and n = 46 for Helm+CCs, and n = 52 for both Helm- and Helm+ TB groups. Two-way ANOVA with Tukey’s multiple comparison test was used for comparing TB groups (both for helminth positive and negative groups) versus CCs (both for helminth negative and positive groups). *, p<0.05; **, p<0.01, ***, p< 0.001.
(PDF)
