# Supplementary material for: Helminth species dependent effects on Th1 and Th17 cytokines in active tuberculosis patients and healthy community controls
Source: PLoS Negl Trop Dis. 2022 Aug 17;16(8):e0010721. doi: 10.1371/journal.pntd.0010721 (PMC9423606; doi:10.1371/journal.pntd.0010721)
Supplement: S1 Fig — PBMCs isolated from TB patients or CCs with or without helminths were stimulated with media alone (Unstim), PPD or SEB and cultured for 19h at 37°C, and PBMC supernatants harvested. The level of IFN-gamma, TNF-alpha, IL-6, IL-17A, IL-10 and IL-4 were determined in plasma and from PBMC supernatants using cytometric bead array. Concentration of IFN-gamma (left) and TNF-alpha (right) (A), IL-6 (left) and IL-17A (right) (B), IL-4 (left) and IL-10 (right). Overall, there were increased levels of pro-inflammatory cytokines in TB patients compared to CCs. Regarding IL-17A, only the Helm-/TB groups show increased levels in plasma whereas Helm+/TB patients were suppressed to the same level as the CC groups (p<0.01). Data are presented as mean ± SEM. n = 24 for Helm-/CCs and n = 46 for Helm+CCs, and n = 52 for both Helm- and Helm+ TB groups. Two-way ANOVA with Tukey’s multiple comparison test was used for comparing TB groups (both for helminth positive and negative groups) versus CCs (both for helminth negative and positive groups). *, p<0.05; **, p<0.01, ***, p< 0.001. (PDF) [file pntd.0010721.s001.pdf]

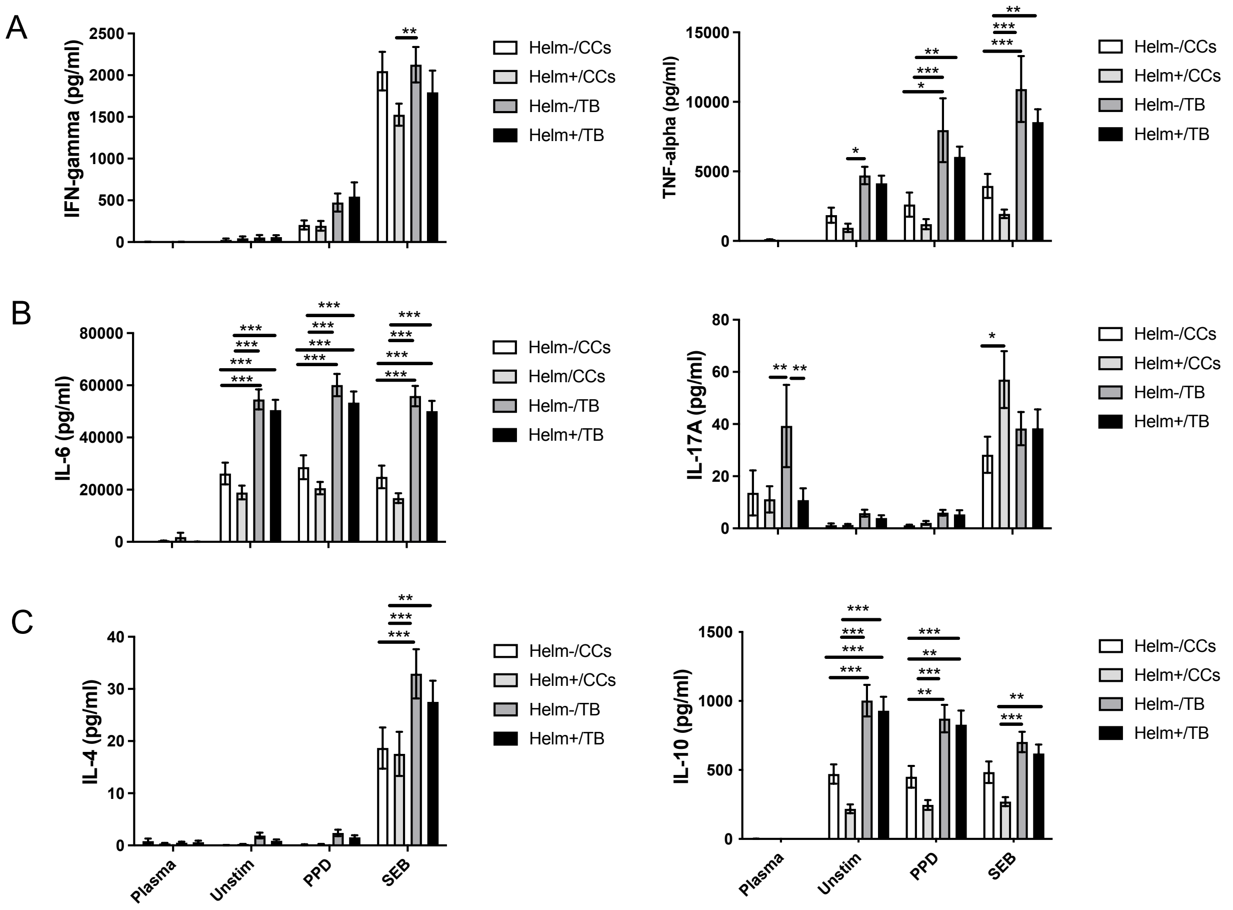

Supporting information S1 Fig. Proinflammatory cytokines and IL-10 are increased in TB patients regardless of helminth infection compared with CCs.

PBMCs isolated from TB patients with or without helminths were stimulated with media alone (Unstim), PPD or SEB and cultured for 19h at 37°C, and PBMC supernatants harvested. The level of IFN-gamma, TNF-alpha, IL-6, IL-17A, IL-10 and IL-4 were determined in plasma and from PBMC supernatants using cytometric bead array. Concentration of IFN-gamma (left) and TNF-alpha (right) (A), IL-6 (left) and IL-17A (right) (B), IL-4 (left) and IL-10 (right). Overall, there were increased levels of pro-inflammatory cytokines in TB patients compared to CCs. Regarding IL-17A, only the Helm-/TB groups show increased levels in plasma whereas Helm+/TB patients were suppressed to the same level as the CC groups ($p<0.01$). Data are presented as mean ± SEM.

n=24 for Helm-/CCs and n=46 for Helm+CCs, and n=52 for both Helm- and Helm+ TB groups. Two-way ANOVA with Tukey's multiple comparison test was used for comparing TB groups (both for helminth positive and negative groups) versus CCs (both for helminth negative and positive groups). *, $p<0.05$; **, $p<0.01$, ***, $p< 0.001$.
